# Association between chronic kidney disease and colorectal cancer: evidence from meta-analysis and Mendelian randomization

**DOI:** 10.1007/s12672-025-02785-9

**Published:** 2025-06-01

**Authors:** Shuyi Qian, Yuting Gong, Ying Chen

**Affiliations:** Department of Nephrology and Laboratory of Kidney Disease, Hunan Clinical Research Center for Chronic Kidney Disease, Hunan Provincial People’s Hospital, Hunan Normal University, No. 61 Jiefang West Road, Furong District, Changsha, 410002 China

**Keywords:** Chronic kidney disease, Colon cancer, Rectal cancer, Colorectal cancer, Meta-analysis, Mendelian randomization

## Abstract

**Objective:**

Observational studies have yielded inconsistent findings on the relationship between chronic kidney disease (CKD) and the risk of colorectal cancer (CRC). This research utilized a meta-analysis of cohort studies along with two-sample Mendelian randomization (MR) approaches to explore the causal impact of CKD on CRC.

**Methods:**

A thorough search was performed across PubMed, Web of Science, Embase, and the Cochrane Library, encompassing all relevant studies from their inception until December 20, 2024. The data on the causal link between CKD and CRC were pooled using risk ratio (RR), with findings reported within 95% confidence interval (CI). For MR analysis, data were synthesized from genome-wide association studies (GWAS) that concentrated on CKD and CRC in cohorts of European descent. The inverse-variance weighted (IVW) approach was utilized as the primary method for MR analysis.

**Results:**

This meta-analysis encompassed 8 cohort studies involving 613,135 CKD patients and 733,068 controls. The pooled results revealed that CKD was associated with an increased risk of CRC in the total population (RR: 1.332, 95% CI 1.084–1.637, 95% prediction interval [PI] = 0.669–2.651). Subgroup analysis indicated that the association between CKD and elevated CRC risk was more pronounced in individuals younger than 50 years (RR: 2.119, 95% CI 1.205–3.725, 95% PI = 0.276–16.284) and in women (RR: 1.550, 95% CI 1.121–2.144, 95% PI = 0.594 to 4.043). Further MR analysis, using the IVW approach, showed a significant causal connection between CKD and the heightened CRC risk (odds ratio [OR]: 1.171, 95% CI 1.063–1.289, *p* = 0.001). Results from the sensitivity analyses provided no evidence of heterogeneity or horizontal pleiotropy in MR analysis.

**Conclusion:**

Evidence from meta-analysis and MR analysis suggested that CKD may increase the risk of CRC. It remains essential to further investigate the relationship between CKD and CRC incidence across diverse ethnic populations.

**Supplementary Information:**

The online version contains supplementary material available at 10.1007/s12672-025-02785-9.

## Introduction

Chronic kidney disease (CKD) represents a major global health issue, impacting roughly 10–15% of the population and ranking among the top causes of mortality worldwide [1, 2]. It is marked by a decline in glomerular filtration rate (GFR) or elevated albuminuria levels [3]. Similarly, cancer is a significant concern, responsible for approximately 10 million deaths annually and standing as the second leading cause of death after cardiovascular disease [4]. Notably, cancer accounts for the majority of non-cardiovascular fatalities in individuals with CKD [5]. As both CKD and cancer prevalence rise with the aging global population, the clinical significance of cancer in CKD patients is expected to grow [6].

Colorectal cancer (CRC) ranks as the third most frequently diagnosed malignancy globally and represents the second leading cause of cancer-related mortality, with over 1.9 million new cases and nearly 904,000 deaths reported worldwide in 2022 [7]. Among individuals with CKD, CRC constitutes the third leading cause of cancer-related mortality in kidney transplant recipients and is also one of the six most frequent cancer-related causes of death in those undergoing dialysis [8]. Notably, outcomes for CKD patients diagnosed with CRC are often worse compared to the general population. Specifically, mortality from CRC is approximately twofold higher in dialysis patients and kidney transplant recipients than in age- and sex-matched general population [5, 9]. 

Numerous studies have identified a heightened likelihood of CRC in kidney transplant recipients and CKD patients when compared to the general population, with standardized incidence ratios (SIRs) ranging from no significant association to a twofold increase [10–13]. A meta-analysis has estimated an overall SIR of 1.18 [14], yet the precise magnitude of CRC risk in CKD patients remains uncertain and insufficiently investigated. The limitations of existing research further complicate this issue. Most observational studies to date have relied on single-cohort designs without appropriate control groups [15–17], making it difficult to effectively account for confounding variables and preventing direct calculation of risk ratios (RRs) or hazard ratios (HRs). Consequently, such studies fall short in fully clarifying the causal link between CKD and CRC. This underscores the need for a robust meta-analysis of cohort studies that incorporate control groups and provide RRs or HRs to better assess the association between CKD and CRC.

Nevertheless, observational studies come with inherent limitations, such as residual confounding and measurement errors arising from inaccurate or invalid data reporting, which can skew research outcomes. Some of these methodological challenges can be addressed through Mendelian randomization (MR) analyses. This approach is a robust tool for inferring causal relationships between exposures and outcomes, utilizing single nucleotide polymorphisms (SNPs) as instrumental variables (IVs) [18]. These genetic variants are allocated randomly at conception, akin to the randomization process in randomized controlled trials (RCTs), thereby mitigating the influence of confounding factors and reverse causation [19]. 

To address the limitations of observational research and leverage the strengths of MR analysis, this study aims to systematically evaluate the association between CKD and CRC through a comprehensive meta-analysis of cohort studies and to investigate the potential causal relationship between CKD and CRC risk using MR analysis based on genome-wide association study (GWAS) data. By integrating these approaches, our study seeks to provide robust evidence to clarify whether CKD is causally associated with CRC risk, ultimately contributing to a better understanding of the underlying mechanisms and informing future prevention strategies.

## Methods

### Study design and search strategy for meta-analysis

This study was meticulously conducted in accordance with the PRISMA and MOOSE guidelines. A comprehensive literature search was executed across PubMed, Embase, the Cochrane Library, and Web of Science, covering all records up to December 20, 2024. The search strategy employed a combination of terms including: (“chronic kidney disease”, “CKD”, “chronic renal disease”, “chronic kidney insufficiency”, “chronic renal insufficiency”, “chronic kidney failure”) AND (“colorectal cancer”, “colon cancer”, “rectal cancer”, “colorectal tumor”, “colorectal neoplasm”, “colorectal carcinoma”). This search was conducted without any temporal or linguistic restrictions. Detailed search methodologies for each database were provided in Supplementary Files S1. Furthermore, we examined citations in relevant review articles to assess their suitability.

### Study selection and data extraction

Studies were eligible for inclusion if they satisfied these criteria: (1) retrospective or prospective cohort studies; (2) the exposure variable was CKD; (3) the outcome of interest was CRC; (4) the research provided an effect estimate, such as a RR, HR or incidence rate ratio (IRR), along with the corresponding 95% confidence interval (CI) for the relationship between CKD and CRC. Studies were excluded if they met any of the following conditions: (1) case–control or cross-sectional studies; (2) research lacking a control group; (3) reviews, case reports, animal studies, clinical trials, and abstracts.

Two independent reviewers documented the data from the selected studies using pre-designed extraction forms. For each study, the following details were captured: the first author, publication year, study design, region, population in the exposure and control groups, sample size and age of the cohort, proportion of male, definition of CRC, and Follow-up duration. Any discrepancies encountered during the screening process were resolved through consensus with a third reviewer.

### Risk of bias assessment

The risk of bias for cohort studies was evaluated using the Newcastle–Ottawa Scale (NOS), which considers three principal criteria: (1) the selection of study groups, (2) the comparability of these groups, and (3) outcome assessment [20]. Each study could receive up to one point for items in the selection and outcome domains, and up to two points in the comparability domain. Overall scores for each study ranged from 0 to 9. Studies were classified as low quality with scores between 0 and 4, moderate quality with scores of 5–6, and high quality with scores of 7–9 [21].

### Statistical analysis for meta-analysis

Given CRC is a rare disease, we approximated the HR and IRR as RR when synthesizing the estimates from various studies [22]. The relationship between CKD and CRC is presented as RR with a 95% CI. Heterogeneity among studies was assessed using Cochran’s Q test, I^2^ statistics, and the 95% prediction interval (PI). As per Higgins et al., heterogeneity can be classified as low, moderate, and high for I^2^ values of 25%, 50%, and 75%, respectively [23]. We utilized random-effects model to pool the RR. Subgroup analysis was conducted to explore heterogeneity sources based on race, gender, age, and types of dialysis. Additionally, sensitivity analysis was performed to mitigate heterogeneity. To evaluate the potential for publication bias, we used funnel plots along with Begg’s and Egger’s tests. All statistical analyses were performed using R software 4.3.2 and STATA 12.0, with tests conducted at a two-sided significance level of 5%.

### Study design for MR analysis

We examined the causal link between CKD and the risk of CRC using a two-sample MR approach. The detailed methodology was depicted in Supplementary Fig. S1. Using summary data from GWAS, we identified SNPs significantly correlated with CKD to act as IVs for estimating the causal impact of the exposure. To ensure the robustness of the MR analysis, three key assumptions were met: (1) IVs must be strongly associated with the exposure, (2) IVs must be independent of confounding variables, and (3) IVs must affect the outcome solely through the exposure [24]. Furthermore, our research adhered to the STROBE-MR reporting guidelines [25]. All data used in this study are publicly available summary statistics from GWAS, obviating the need for additional ethical approval or informed consent.

### Data sources and instrumental variable selection

CKD data were sourced from the FinnGen research consortium (https://r11.finngen.fi/), which included 11,265 CKD cases and 436,208 control subjects. Summary data for CRC were derived from a recent large-scale GWAS involving 6,581 CRC cases and 463,421 controls of European ancestry [26]. Supplementary Table S1 provided detailed information on the datasets used.

To identify SNPs significantly associated with CKD as valid IVs, we used a genome-wide significance level of *p* < 5 × 10^–8^. We further refined our selection by removing SNPs with a linkage disequilibrium r^2^ > 0.001 and those within 10,000 kb to minimize linkage disequilibrium effects. The validity of the IVs was evaluated using the *F* statistic, with an *F*-value exceeding 10 indicating strong instrument strength and mitigating the risk of weak instrument bias [27]. To address horizontal pleiotropy, we utilized the GWAS catalog (https://www.ebi.ac.uk/gwas/) to identify SNP associations with confounders, applying a significance threshold of *p* < 5 × 10^–8^. This approach ensured the removal of SNPs that could confound the analysis. Finally, we combined data from both exposure and outcome, excluding any palindromic sequences to maintain consistency in allele effects.

### MR analysis

In our MR analysis, we utilized the inverse-variance weighted (IVW) approach, an extension of the Wald ratio estimator based on meta-analysis principles [28]. This approach yields the most precise estimates under the assumption that all SNPs are valid instruments, though it is susceptible to pleiotropic effects [29]. To enhance the robustness of our findings, we also applied the MR-Egger, weighted median, weighted mode and simple mode methods [30, 31]. These five strategies are considered among the most robust and commonly used in MR studies, providing strong analytical capabilities. The weighted median approach necessitates that at least 50% of the SNPs are valid IVs [31], whereas the MR-Egger method can produce unbiased estimates even in the presence of pleiotropy among all selected IVs [30]. *P*-values below 0.05 were considered indicative of statistical significance.

### Sensitivity analysis

To further confirm the robustness of our results and evaluate pleiotropy, we performed a series of sensitivity analyses. Heterogeneity was assessed using Cochrane’s Q test, with IVs showing a *p*-value below 0.05 deemed heterogeneous [32]. We employed the MR-Egger intercept and the MR pleiotropy residual sum and outlier (MR-PRESSO) method to identify potential horizontal pleiotropy [33, 34]. When pleiotropy was detected, we repeated the MR analyses after removing outliers. The stability of our findings was further scrutinized using the leave-one-out method, which evaluates the impact of each individual SNP on the results. Scatter plots and forest plots were employed to visualize the association between CKD and CRC. Additionally, funnel plot analysis was conducted to reinforce the robustness of our results. All statistical analyses were performed using R software 4.3.2, utilizing the “TwoSampleMR” and “MendelianRandomization” packages.

## Results

### Meta-analysis results

#### Study selection

**Fig. 1:**
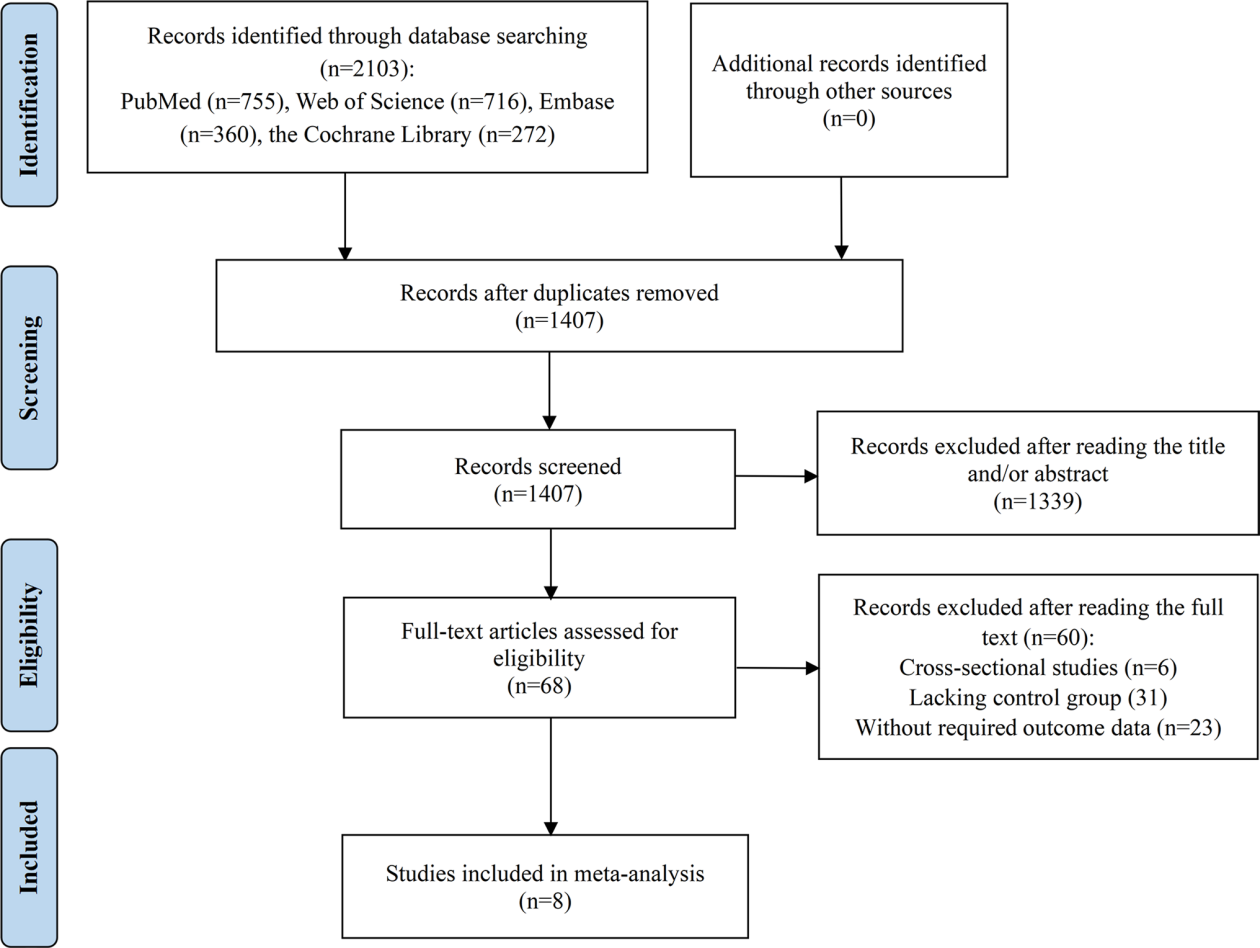
Flow diagram of the process of study selection in meta-analysis

Initially, a total of 2,103 studies were retrieved from various databases. Following the removal of 2,035 studies due to duplication or failure to meet inclusion criteria, 68 studies remained for full-text evaluation. After a comprehensive assessment, 60 studies were deemed unsuitable for inclusion: 6 were cross-sectional in nature; 31 lacked a control group; and 23 did not provide the necessary outcome data. Ultimately, 8 studies met the criteria for quantitative synthesis [35–42]. The detailed selection process were depicted in Fig. 1.

#### Study characteristics

The included 8 articles were retrospective cohort studies, involving a total of 613,135 CKD patients and 733,068 controls. Notably, all studies were conducted in East Asia. Each cohort included in the analysis comprised a substantial sample size, ranging from 17,850 to 943,516 participants. Moreover, the gender distribution within the cohorts was relatively balanced, with no significant overrepresentation of either male or female participants. The definition of CRC in all included studies was consistently based on International Classification of Diseases, Ninth Revision, Clinical Modification (ICD-9-CM) or ICD-10 codes. Detailed characteristics of the included studies were provided in Table 1.

**Table 1:** Summary of the characteristics of included studies

First author (year)	Study design	Region	CKD cohort	Control cohort	Sample size (E/C)	Proportion of male (E/C)	Age (E/C, year)	Definition of CRC	Follow-up duration (E/C, year)	NOS
Kwon (2019)	RCS	Korea	ESRD patients who underwent dialysis	Healthy individuals and non-dialysis subjects	48,315/48,315	56.3%/57.2%	0 to ≥ 80	ICD-10 codes	3.8 ± 2.6/5.0 ± 2.7	8
Lee (2018)	RCS	China	ESRD patients who underwent PD or HD	Propensity score-matched subjects without dialysis	10,107/16,845	PD: 44.4%/HD: 46.7%/Comparison: 45.5%	≥ 30	ICD-9-CM codes	NR	7
Lin (2015)	RCS	China	CKD patients who underwent long-term dialysis	Subjects who never received dialysis	47,037/47,037	47.2%/47.2%	58.2 ± 15.1/57.0 ± 15.4	ICD-9-CM codes	3.0 ± 2.1/Median (IQR): 2.5 (1.2–4.4)	8
Wu (2013)	RCS	China	Adult patients who had a diagnosis of CKD	Subjects without CKD	15,975/79,875	52.0%/52.0%	≥ 18	ICD-9-CM codes	3	8
Park (2019)	RCS	Korea	Patients with pre-dialysis CKD	Individuals without any CKD indicative laboratory results	471,758/471,758	51.5%/51.5%	Median (IQR): 64 (55–71)	ICD-10 codes	4.77 (Median)/4.80 (Median)	8
Jung (2022)	RCS	Korea	CKD patients who received kidney transplantation	Individuals who had not received kidney transplantation	12,634/12,634	65.8%/48.1%	≥ 20/ ≥ 20 (99.9%)	ICD-10 codes	2.9 (Median)/5.0 (Median)	8
Chung (2012)	RCS	China	ESRD patients who underwent PD, HD or renal transplantation	Subjects without ESRD	3,570/14,280	49.2%/49.2%	61.3 ± 14.9/60.9 ± 15.0	ICD-9-CM codes	3.18 (Mean)/4.45 (Mean)	8
Wang (2019)	RCS	China	ESRD patients who received kidney transplantation	Subjects without kidney transplantation	3,739/42,324	40.97%/45.05%	61.84 ± 12.63/52.31 ± 14.74	ICD-9-CM codes	NR	7

*CKD* chronic kidney disease, *E* Exposure group, *C* control group, *CRC* colorectal cancer, *NOS* Newcastle–Ottawa Scale, *RCS* retrospective cohort study, *ESRD* end-stage renal disease, *ICD* International Classification of Diseases, *PD* peritoneal dialysis, *HD* hemodialysis, *CM* Clinical Modification, *NR* not reported, *IQR* interquartile range

#### Risk of bias in studies

We assessed the risk of bias in the included cohort studies using the the NOS quality assessment tool. All included studies were rated as high quality, with scores above 7. The primary limitations identified were the lack of control for secondary confounding factors and insufficient follow-up duration in certain studies. The quality assessment details for the included studies were presented in Supplementary Table S2.

#### Overall and subgroup analysis of the association between CKD and CRC

**Fig. 2:**
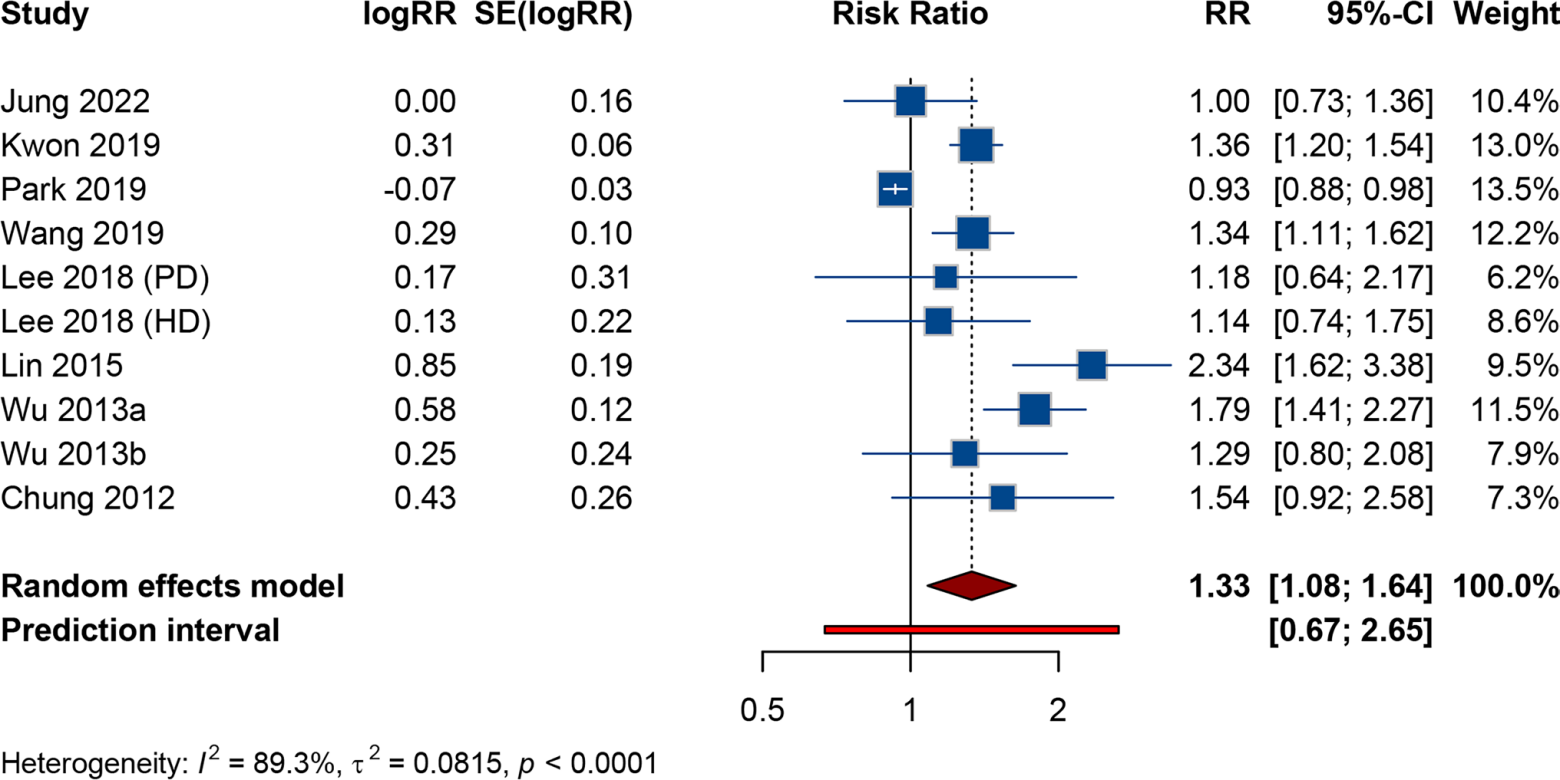
Meta-analysis of the causal association between chronic kidney disease and colorectal cancer

The results of the meta-analysis and subgroup analysis on the association between CKD and CRC were presented in Table 2. The aggregated findings from the random-effects model (I^2^ = 89.3%, Tau^2^ = 0.0815) revealed that CKD was associated with an increased risk of CRC (RR: 1.332, 95% CI 1.084–1.637, 95% PI = 0.669–2.651) (Fig. 2). Since all included studies were conducted in East Asia, subgroup analyses stratified by race yielded consistent results in East Asian populations, showing that patients with CKD had a significantly increased risk of developing CRC (RR: 1.332, 95% CI 1.084–1.637, 95% PI = 0.669–2.651) (Supplementary Fig. S2). Furthermore, among individuals younger than 50 years, CKD was associated with an elevated risk of CRC (RR: 2.119, 95% CI 1.205–3.725, 95% PI = 0.276–16.284) (Supplementary Fig. S3A); however, no significant association was observed in individuals aged 50 years or older (RR: 1.253, 95% CI 0.955–1.644, 95% PI = 0.557–2.818) (Supplementary Fig. S3B). Similarly, No significant association was identified in males (RR: 1.482, 95% CI 0.971–2.264, 95% PI = 0.363–6.049) (Supplementary Fig. S4A), whereas CKD was found to increase the risk of CRC in females (RR: 1.550, 95% CI 1.121–2.144, 95% PI = 0.594 to 4.043) (Supplementary Fig. S4B). Additionally, subgroup analyses based on dialysis modality revealed that the risk of CRC was not increased in CKD patients undergoing either hemodialysis or peritoneal dialysis (all *p* > 0.05) (Supplementary Fig. S5A-5B).

**Table 2:** Pooled effect and subgroup analysis of the causal association between chronic kidney disease and colorectal cancer

Outcomes and subgroups	Number of studies	Meta-analysis				Heterogeneity	
		RR	95% CI	*p* value	95% PI	I^2^, Tau^2^	*p* value
Overall	8	1.332	1.084–1.637	0.007	0.669–2.651	89.3%, 0.0815	< 0.001
Race							
East Asian	8	1.332	1.084–1.637	0.007	0.669–2.651	89.3%, 0.0815	< 0.001
Age							
< 50 years	3	2.119	1.205–3.725	0.009	0.276–16.284	57.1%, 0.1418	0.097
≥ 50 years	3	1.253	0.955–1.644	0.104	0.557–2.818	70.0%, 0.0661	0.010
Sex							
Male	4	1.482	0.971–2.264	0.068	0.363–6.049	81.2%, 0.1485	0.001
Female	4	1.550	1.121–2.144	0.008	0.594–4.043	60.3%, 0.0634	0.056
Dialysis type							
Hemodialysis	2	1.275	0.913–1.781	0.154	0.146–11.129	0%, 0	0.411
Peritoneal dialysis	2	1.201	0.670–2.153	0.538	0.027–52.786	0%, 0	0.846

#### Sensitivity analysis and publication bias

In the sensitivity analysis, we calculated pooled RR and their 95% CI by sequentially excluding individual studies to evaluate each study’s impact on the overall results. This approach indicated that the omission of any single study did not significantly impact the quantitative findings, underscoring the robustness and reliability of the combined results (Supplementary Fig. S6). Begg’s and Egger’s tests suggested the presence of potential publication bias in the causal relationship between CKD and CRC (Begg’s test: *p* = 0.592, Egger’s test: *p* = 0.032). Further trim-and-fill method was applied to correct for publication bias. The comparison between the adjusted and original results revealed no differences, affirming the reliability of the findings. The funnel plot was depicted in Supplementary Fig. S7.

### Mendelian randomization analysis

#### Selection of instrumental variables

Applying a GWAS significance threshold of *p* < 5 × 10^–8^ and eliminating SNPs in linkage disequilibrium, we initially pinpointed 5 SNPs linked to CKD to serve as IVs. No SNPs were excluded due to significant associations with confounding factors. Thus, a final set of 5 SNPs for CRC was employed as IVs in the MR analysis. The robustness of these SNPs was validated by *F*-statistics exceeding 10, suggesting a minimal risk of weak instrument bias. Detailed information on these SNPs can be found in Supplementary Tables S3-4.

#### MR analysis of CKD on CRC

**Fig. 3:**
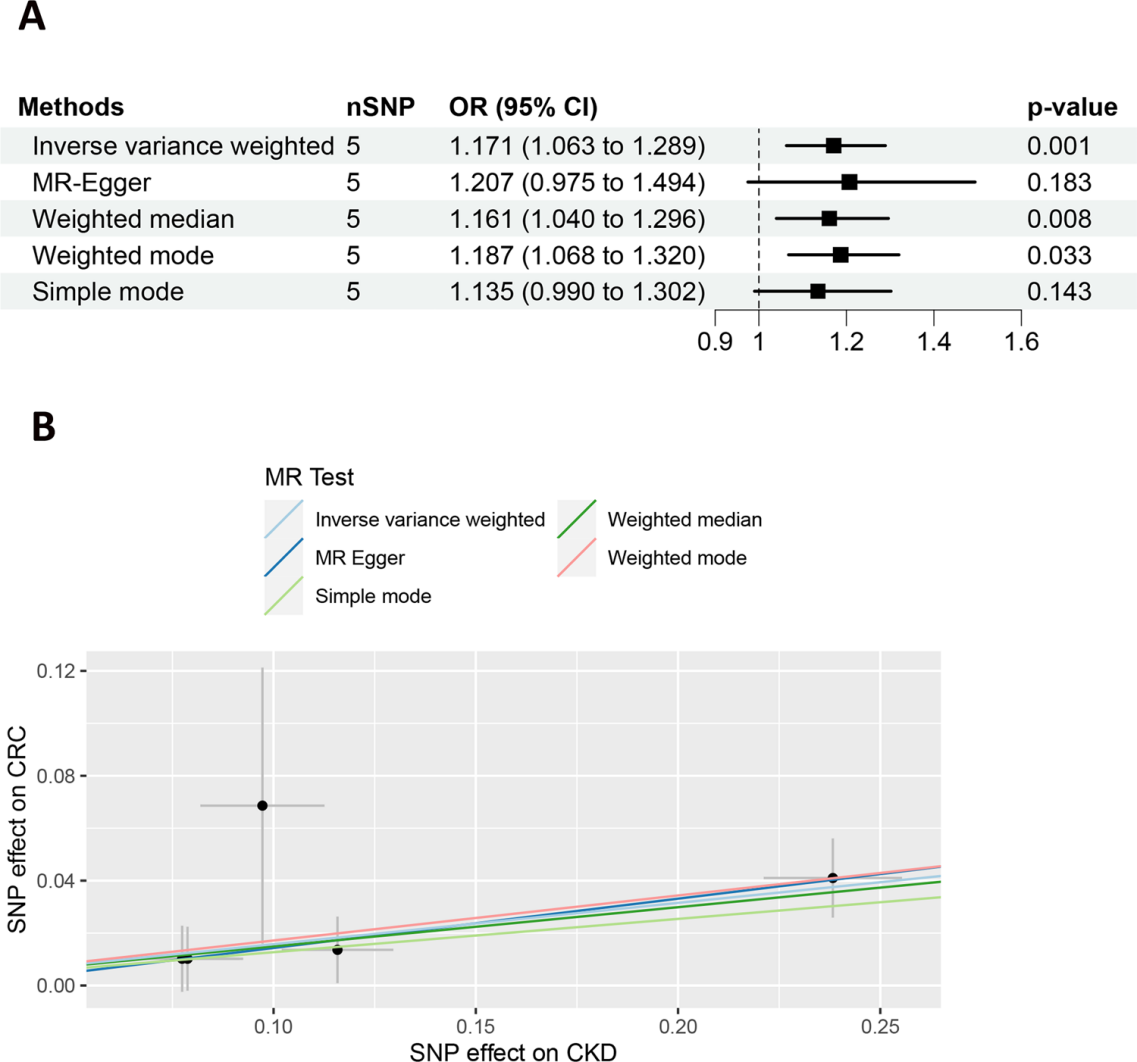
MR analysis and scatter plots of the causal effect of chronic kidney disease (CKD) on colorectal cancer (CRC). **A** MR analysis of the causal effect of CKD on CRC. **B** Scatter plots of the causal effect of CKD on CRC

In our MR analysis, we investigated the relationship between CKD and CRC, with the results depicted in Fig. 3A. Employing the IVW method, we detected a significant positive causal association between CKD and CRC (odds ratio [OR]: 1.171, 95% CI 1.063–1.289, *p* = 0.001). This significant relationship was further supported by additional MR analyses using both the weighted median (OR: 1.161, 95% CI 1.040–1.296, *p* = 0.008) and weighted mode (OR: 1.187, 95% CI 1.068–1.320, *p* = 0.033) techniques. Furthermore, Fig. 3B presented scatter plots with MR intercepts close to zero, suggesting minimal horizontal pleiotropy effects. The forest plots showed the estimated causal effects between CKD and CRC (Supplementary Fig. S8).

#### Sensitivity analyses

Heterogeneity evaluations were performed, with Cochrane’s Q statistics yielding *p*-values greater than 0.05 (*p* = 0.867), indicating no evidence of heterogeneity among the IVs. Additionally, the MR-Egger regression intercept, which assesses potential horizontal pleiotropy, did not reveal any significant pleiotropic effects (*p* = 0.775). These findings are corroborated by the MR-PRESSO analysis, which similarly did not identify any notable outliers for horizontal pleiotropy (*p* = 0.917). A leave-one-out analysis, used to gauge the influence of individual SNP, showed no substantial impact on the overall conclusion (Supplementary Fig. S9). Additionally, funnel plots provided in Supplementary Fig. S10 confirmed the absence of substantial bias, further validating the robustness of our findings.

## Discussion

This study is the first to investigate the causal relationship between CKD and CRC at both population and genetic scales through meta-analysis and MR method. Through a comprehensive meta-analysis that included the most recent cohort studies with matched control groups, we found that CKD patients faced a markedly higher risk of developing CRC compared to those without CKD. However, since all included studies were conducted in East Asia, our meta-analysis findings are specific to this population and may not be generalizable to others. Additionally, leveraging data from the GWAS and FinnGen databases, our MR analysis provided robust evidence supporting a significant causal relationship between CKD and an elevated likelihood of CRC occurrence.

Recent research on the relationship between CKD and CRC has yielded inconsistent findings. A Swedish cohort study analyzed cancer incidence among 7,952 CKD patients who underwent kidney transplantation between 1970 and 2008, reporting a cumulative cancer incidence of 12% within 20 years post-transplantation. While the SIR for breast and prostate cancer was not significantly elevated, the SIR for colon cancer reached 2.3 [43]. Similarly, a study from China found that renal transplant recipients faced approximately double the risk of CRC compared to the general population [10]. Additionally, Butler et al. [44] observed a 1.3-fold increase in the SIR for CRC among patients with end-stage renal disease (ESRD) undergoing dialysis. Another large-scale cohort study reported a relative risk of 3.3 for advanced colorectal adenomas in kidney transplant recipients compared to healthy controls [45]. A recent study examining the incidence of CRC among hemodialysis patients who underwent kidney transplantation identified a 1.34-fold increase in the cumulative incidence of CRC in ESRD patients following transplantation compared to those who did not receive a transplant [41]. Nevertheless, other recent investigations have failed to corroborate these findings, instead reporting no significant difference in CRC incidence between kidney transplant recipients and the general population [9, 12]. Unlike most previous studies, which lacked matched control groups and predominantly relied on SIR estimates, our study conducted a pooled analysis of cohort studies with comparable control groups, reducing the impact of confounding variables and yielding more precise estimates of the CRC risk associated with CKD. Notably, all studies included in our meta-analysis were conducted in East Asia, providing strong evidence for an elevated CRC risk among CKD patients within East Asian populations. Furthermore, our MR analysis, using genetic data from individuals of European ancestry, confirmed the causal association between CKD and the increased risk of CRC. However, the possible causal relationship between CKD and CRC in other populations remains unclear and warrants further exploration.

The exact biological mechanisms underlying the elevated risk of CRC in patients with CKD remain unclear. However, previous studies suggest a link to immunosuppression and chronic inflammation. Immunosuppression may weaken the immune system’s ability to prevent tumorigenesis and allow oncogenic viruses to reactivate [46, 47]. Prolonged use of calcineurin inhibitors is identified as a significant risk factor for post-transplant malignancies [48, 49], potentially promoting tumor development through mutations leading to adenoma-to-carcinoma progression [49, 50]. Additionally, some research indicated that immunosuppressive regimens may enhance systemic inflammation, causing immune system imbalances and increased DNA damage. This is achieved through the upregulation of tumor growth factor-β and vascular endothelial growth factor, alongside changes in gut microbiota, all of which may contribute to CRC development [51, 52]. A previous retrospective study has found a relationship between long-term immunosuppressive therapy and the emergence of colorectal neoplasms [45]. Thus, immunosuppression associated with CKD may be one of the contributing factors to the increased risk of CRC. Moreover, CKD is also associated with elevated proinflammatory cytokine levels [53]. Chronic inflammation is implicated in various cancers, as it can induce mutations, adaptive responses, resistance to apoptosis, and promote angiogenesis [54]. Furthermore, Yu et al. suggested that advanced kidney disease might damage the intestinal lining, leading to malignant transformations [55]. Hence, chronic inflammation and intestinal wall damage associated with CKD may also contribute to the progression of CRC.

Notably, our subgroup analysis identified a markedly elevated risk of CRC in CKD patients under 50 years of age, whereas no significant association was observed in individuals aged 50 years or older. This age-specific disparity aligns with prior studies reporting a 2- to 2.5-fold increase in CRC risk among kidney transplant recipients, with the most pronounced risk evident in younger patients [41, 56]. For instance, the SIR for younger individuals was reported at 13.5, while it declined to less than 3 in patients aged 55 years or older when compared to the age- and sex-matched general population [57]. Moreover, subgroup analyses based on sex revealed a significant association between CKD and heightened CRC risk in women, with no corresponding association detected in men. These findings suggest that younger or female CKD patients may require closer clinical monitoring for CRC compared to their older or male counterparts. However, due to the limited number of studies included in these subgroup analyses, the results should be interpreted with caution. Further research is needed to confirm these observations, particularly through larger and more comprehensive studies. Additionally, the integration of GWAS data in future research could facilitate age- and sex-specific MR analyses to provide deeper insights into these associations.

Our study demonstrates a correlation between CKD and an elevated risk of CRC, underscoring the necessity of recognizing CKD as a significant risk factor in CRC prevention strategies. This evidence suggests that personalized CRC screening protocols for CKD patients may be advantageous. Current CRC screening guidelines primarily focus on age and family history [58], but our findings support the inclusion of CKD as a potential risk factor warranting earlier or more frequent screening. For example, non-invasive methods like fecal immunochemical tests (FIT) could be safer alternatives to colonoscopy for these patients [59]. Nonetheless, practical issues such as cost, patient adherence, and the safety of screening methods need attention. Future research should investigate the cost-effectiveness and feasibility of targeted screening for CKD patients and explore the biological mechanisms linking CKD and CRC.

This study has several limitations that warrant consideration. First, significant heterogeneity was detected among the studies included in the meta-analysis, particularly concerning the dialysis modalities (e.g., hemodialysis and peritoneal dialysis), which may contribute to heterogeneity. Nevertheless, the I^2^ values were within an acceptable range due to the application of random-effects models, which address heterogeneity and provide more conservative estimates. Second, due to insufficient data reported in the included studies, we were unable to perform subgroup analysis stratified by CKD stage, severity, or cause. Third, our MR analysis was restricted to individuals of European descent, which limits the applicability of our findings to other ethnic groups. Similarly, the meta-analysis included only populations from East Asia. Thus, further confirmation of our results in diverse ethnic groups is necessary. Future meta-analyses should prioritize the inclusion of studies from underrepresented populations, such as those from Europe, Africa, and the Americas, to achieve a more thorough understanding of the CKD-CRC relationship across diverse ethnic groups. Likewise, future MR studies ought to utilize genetic information from diverse biobanks, such as the UK Biobank, the All of Us Research Program, and other international cohorts, to determine if the observed relationship is consistent across different ethnic backgrounds.

## Conclusion

In conclusion, our meta-analysis indicated a significant association between CKD and a heightened risk of CRC. Subgroup analyses showed that this association was stronger in individuals under 50 and in women. Further MR analysis with European ancestry GWAS data supported a causal connection between CKD and increased CRC risk. Future research should include diverse ethnic groups in pooled cohort analyses and MR studies using non-European GWAS data to validate the CKD-CRC relationship across populations. Additionally, studies should investigate biological mechanisms and integrate genetic, environmental, and lifestyle factors to develop personalized prevention strategies for CRC.

## Supplementary Information

Supplementary material 1: Fig. S1 Study design diagram and three assumptions of Mendelian randomization. SNPs, single nucleotide polymorphisms; LD, linkage disequilibrium. Fig. S2 Subgroup analysis of the association between chronic kidney disease and colorectal cancer based the race of participants (Subgroup = East Asian). Fig. S3 Subgroup analysis of the association between chronic kidney disease and colorectal cancer based on the age of participants. A < 50 years. B ≥ 50 years. Fig. S4 Subgroup analysis of the association between chronic kidney disease and colorectal cancer based on the gender of participants. A Male. B Female. Fig. S5 Subgroup analysis of the association between chronic kidney disease and colorectal cancer based on the dialysis type. A Hemodialysis. B Peritoneal dialysis. Fig. S6 Sensitivity analysis of the association between chronic kidney disease and colorectal cancer. Fig. S7 Funnel plot of the results from meta-analysis of the association between chronic kidney disease and colorectal cancer. Fig. S8 Forest plots of the results from MR analysis of chronic kidney disease on colorectal cancer. Fig. S9 Leave-one-out analysis of the results from MR analysis of chronic kidney disease on colorectal cancer. Fig. S10 Funnel plots of the results from MR analysis of chronic kidney disease on colorectal cancer.

Supplementary material 2.

Supplementary material 3.

Supplementary material 4.

## Data Availability

Data is provided within the manuscript or supplementary information files.
