# Supplementary material for: Association between chronic kidney disease and colorectal cancer: evidence from meta-analysis and Mendelian randomization
Source: Discov Oncol. 2025 Jun 1;16:974. doi: 10.1007/s12672-025-02785-9 (PMC12127261; doi:10.1007/s12672-025-02785-9)

**Fig. S1** Study design diagram and three assumptions of Mendelian randomization. SNPs, single nucleotide polymorphisms; LD, linkage disequilibrium.


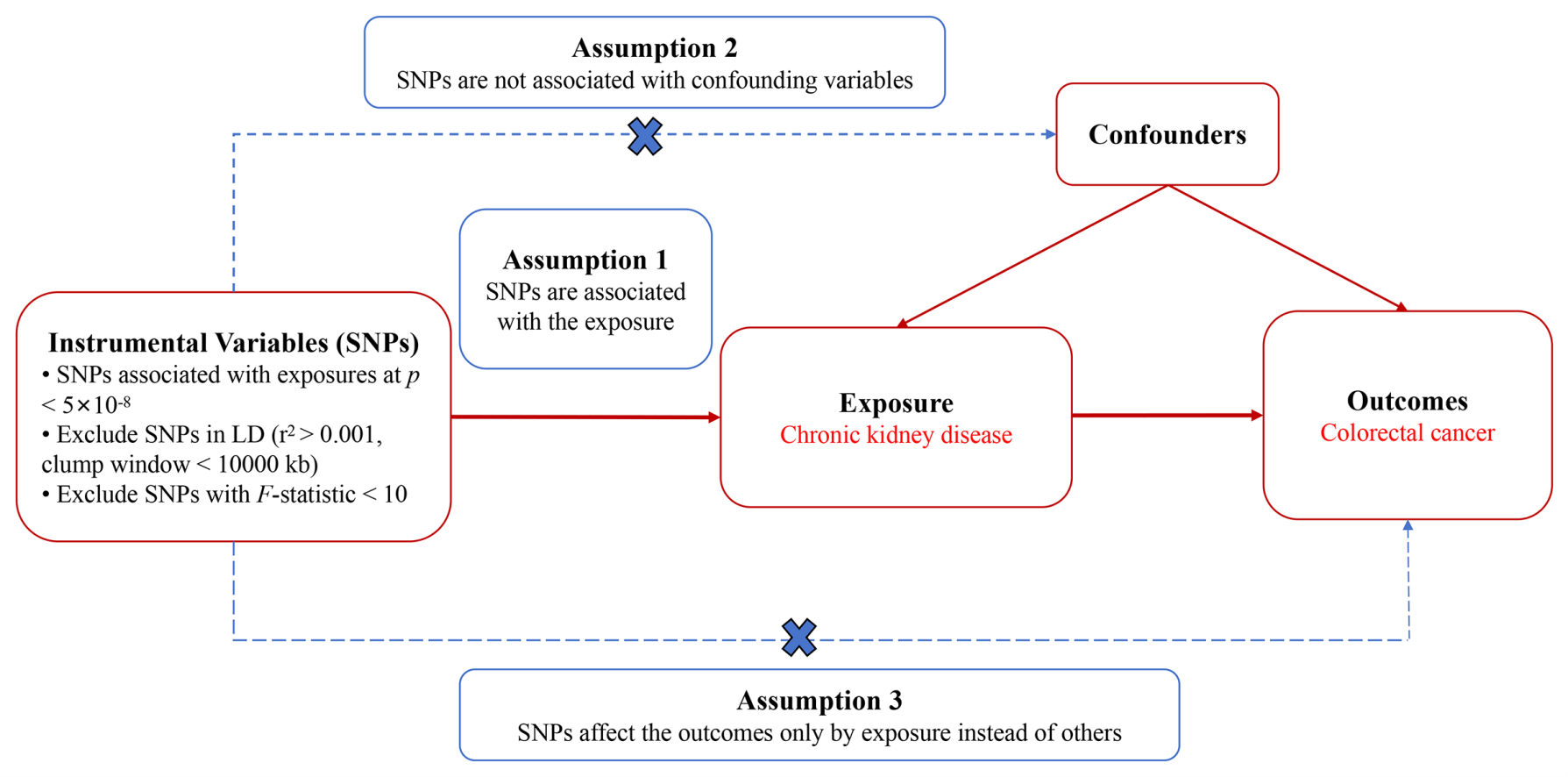


**Fig. S2** Subgroup analysis of the association between chronic kidney disease and colorectal cancer based the race of participants (Subgroup = East Asian).


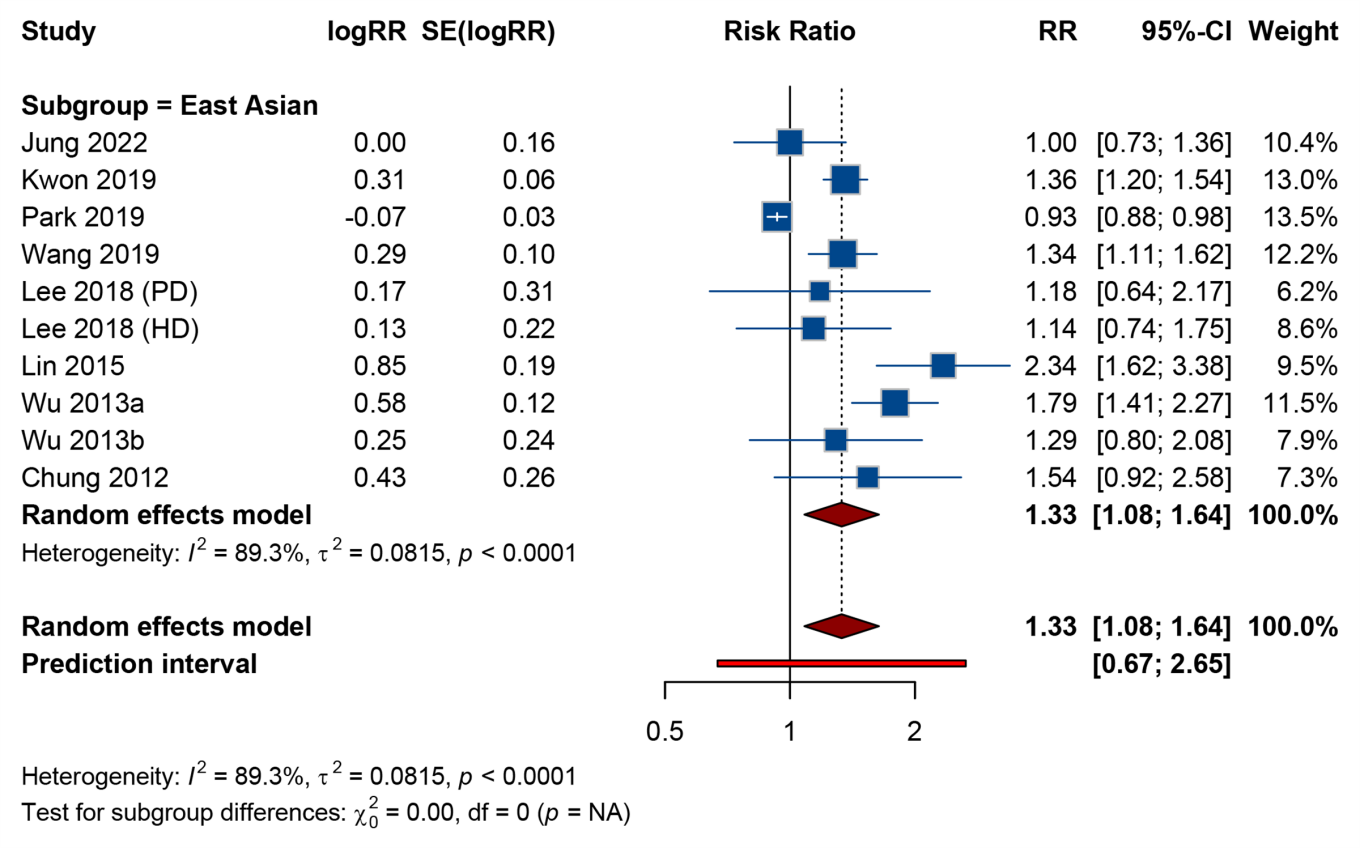


**Fig. S3** Subgroup analysis of the association between chronic kidney disease and colorectal cancer based on the age of participants. (A) < 50 years; (B) ≥ 50 years.


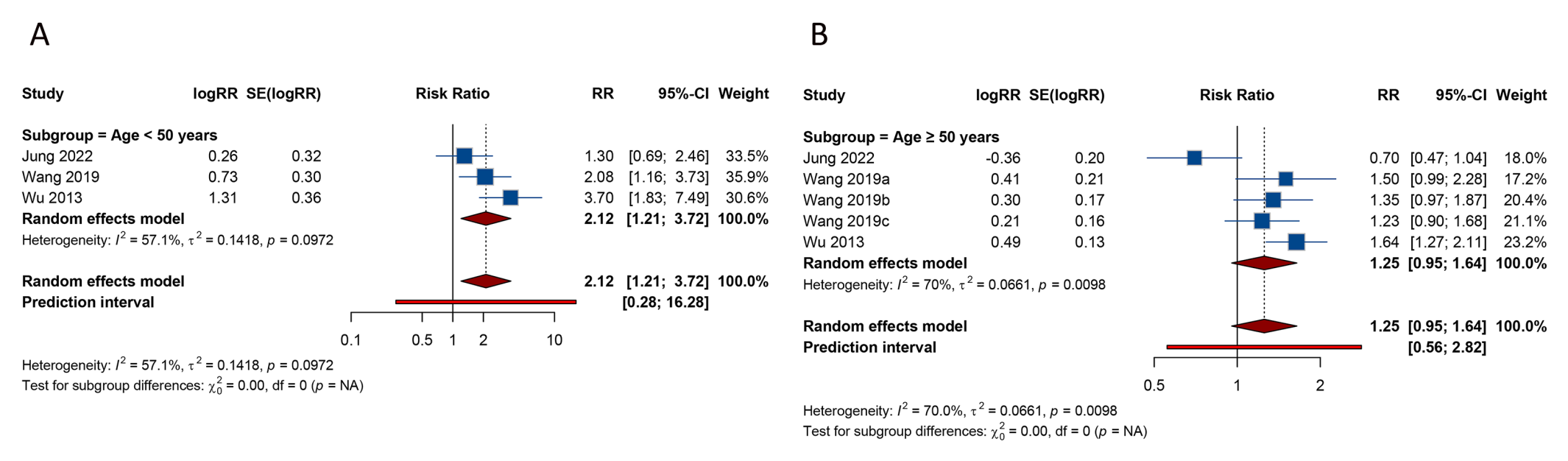


**Fig. S4** Subgroup analysis of the association between chronic kidney disease and colorectal cancer based on the gender of participants. (A) Male; (B) Female.


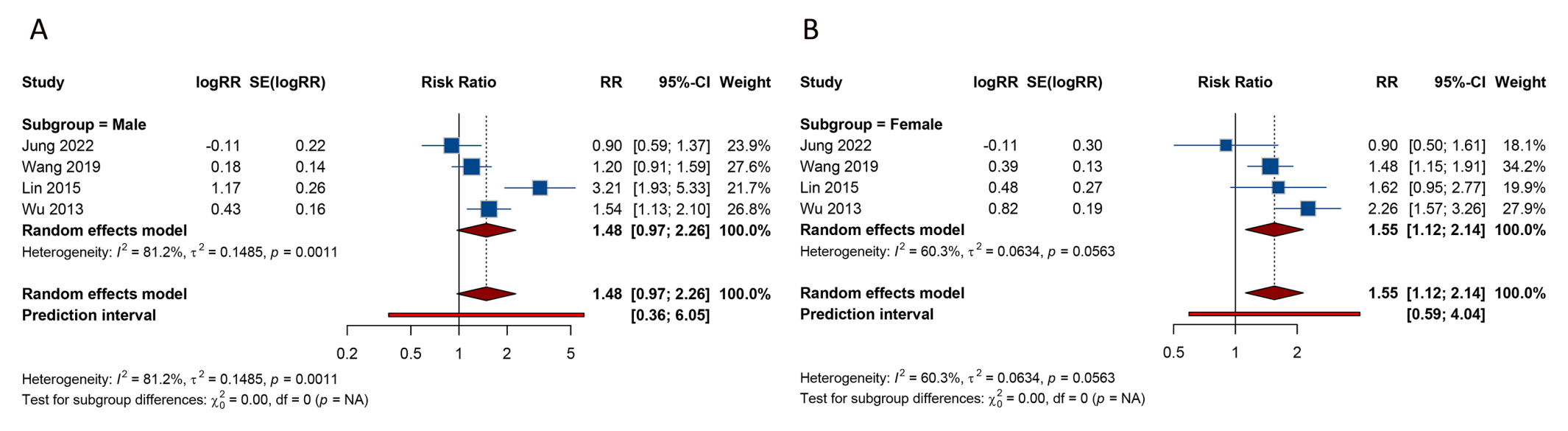


**Fig. S5** Subgroup analysis of the association between chronic kidney disease and colorectal cancer based on the dialysis type. (A) Hemodialysis; (B) Peritoneal dialysis.


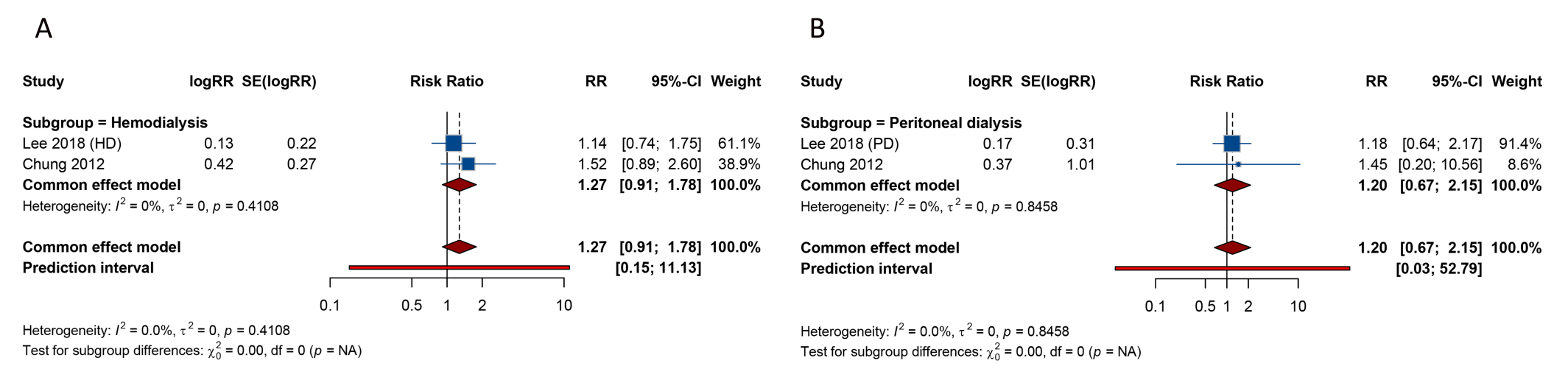


**Fig. S6** Sensitivity analysis of the association between chronic kidney disease and colorectal cancer.


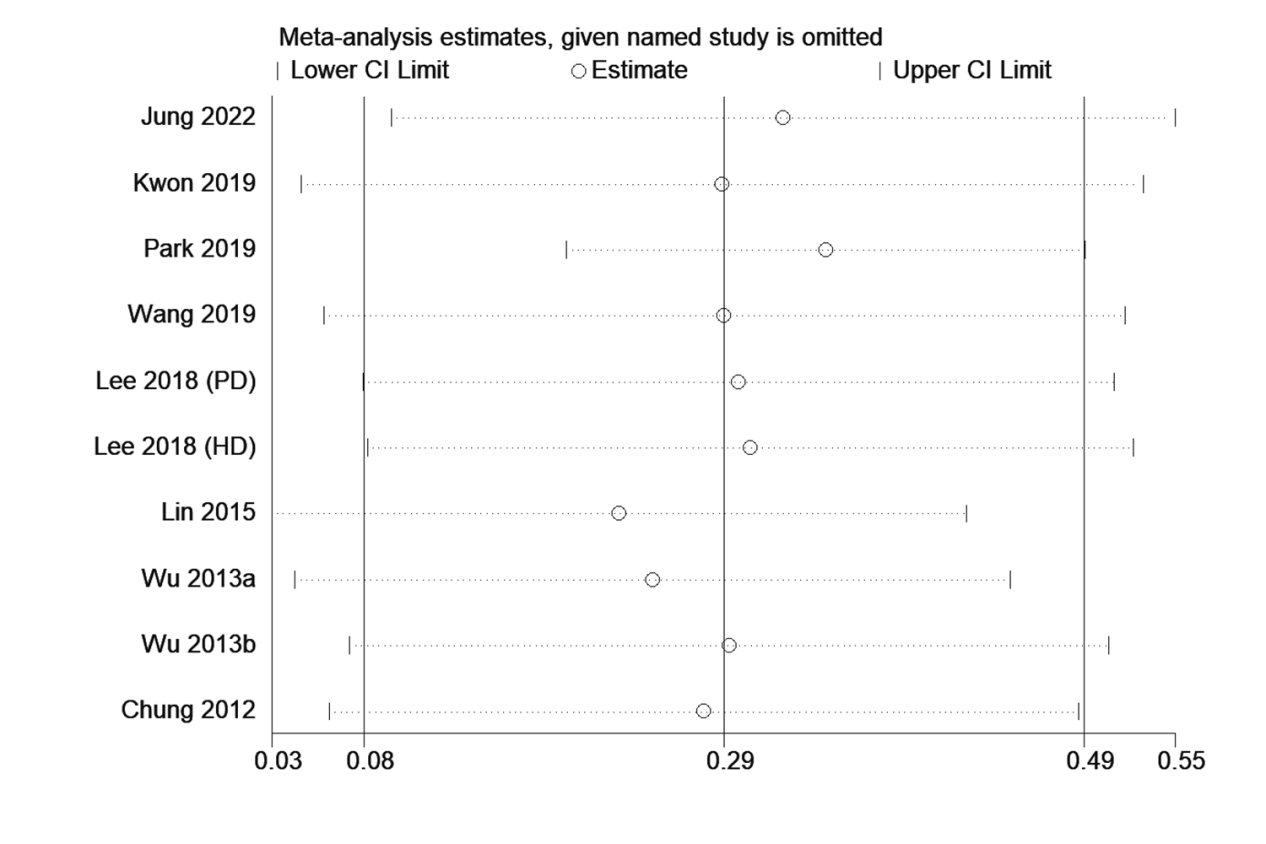


**Fig. S7** Funnel plot of the results from meta-analysis of the association between chronic kidney disease and colorectal cancer.


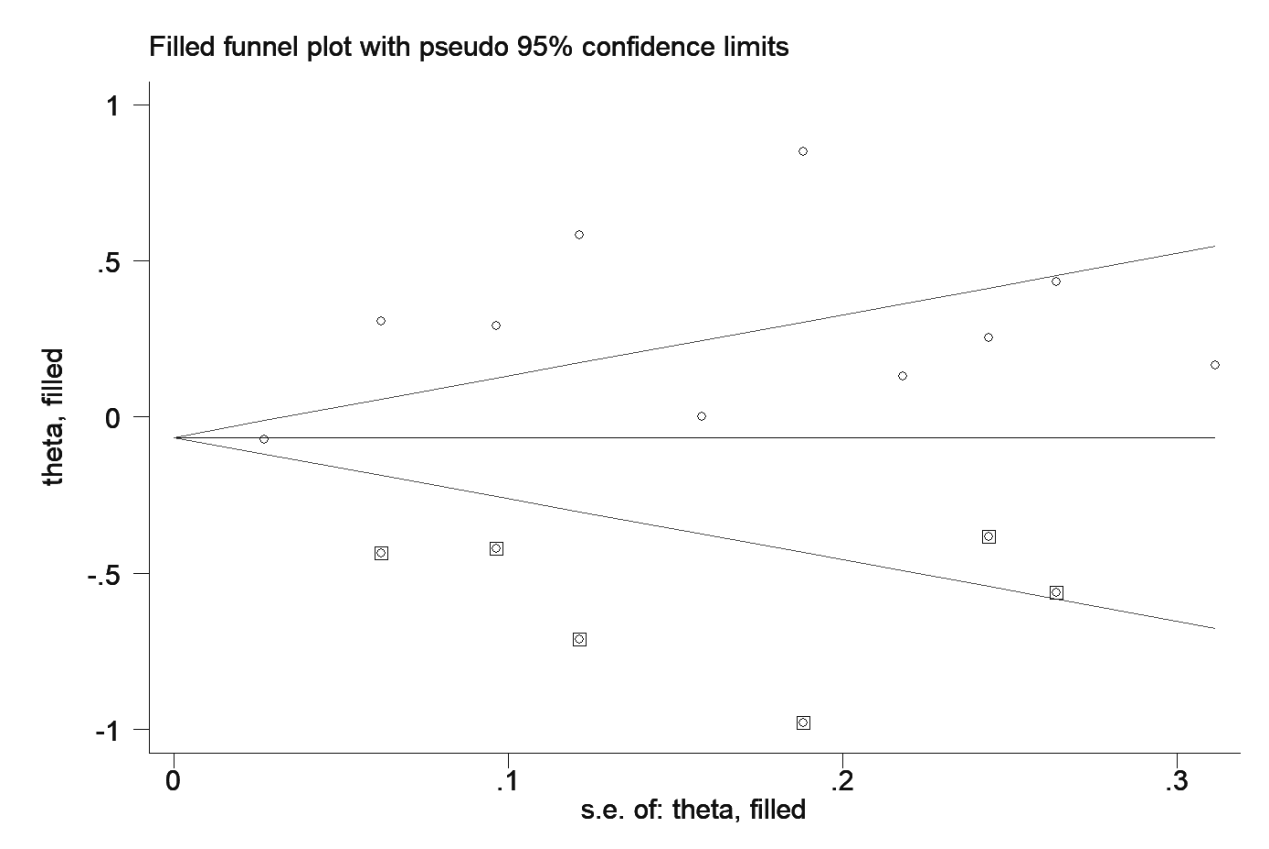


**Fig. S8** Forest plots of the results from MR analysis of chronic kidney disease on colorectal cancer.


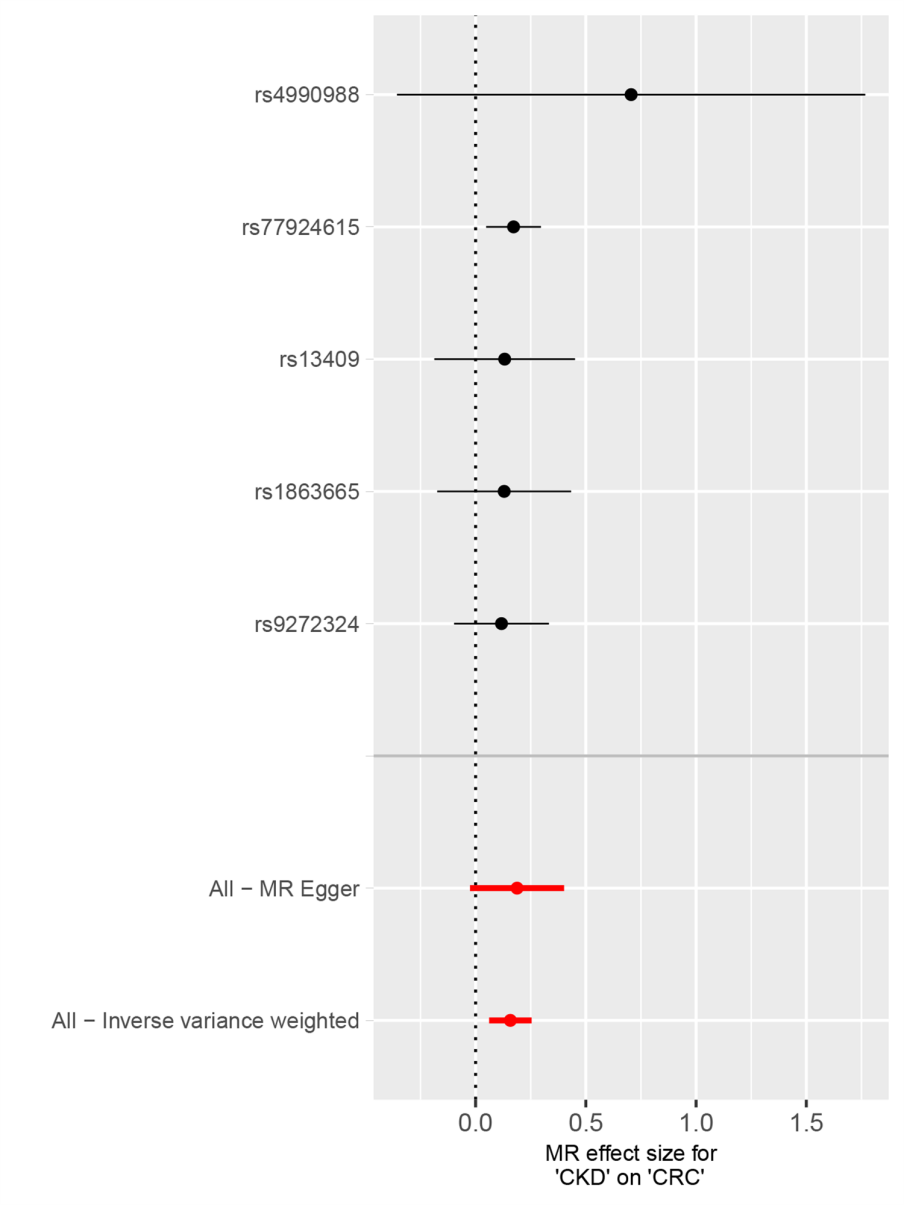


**Fig. S9** Leave-one-out analysis of the results from MR analysis of chronic kidney disease on colorectal cancer.


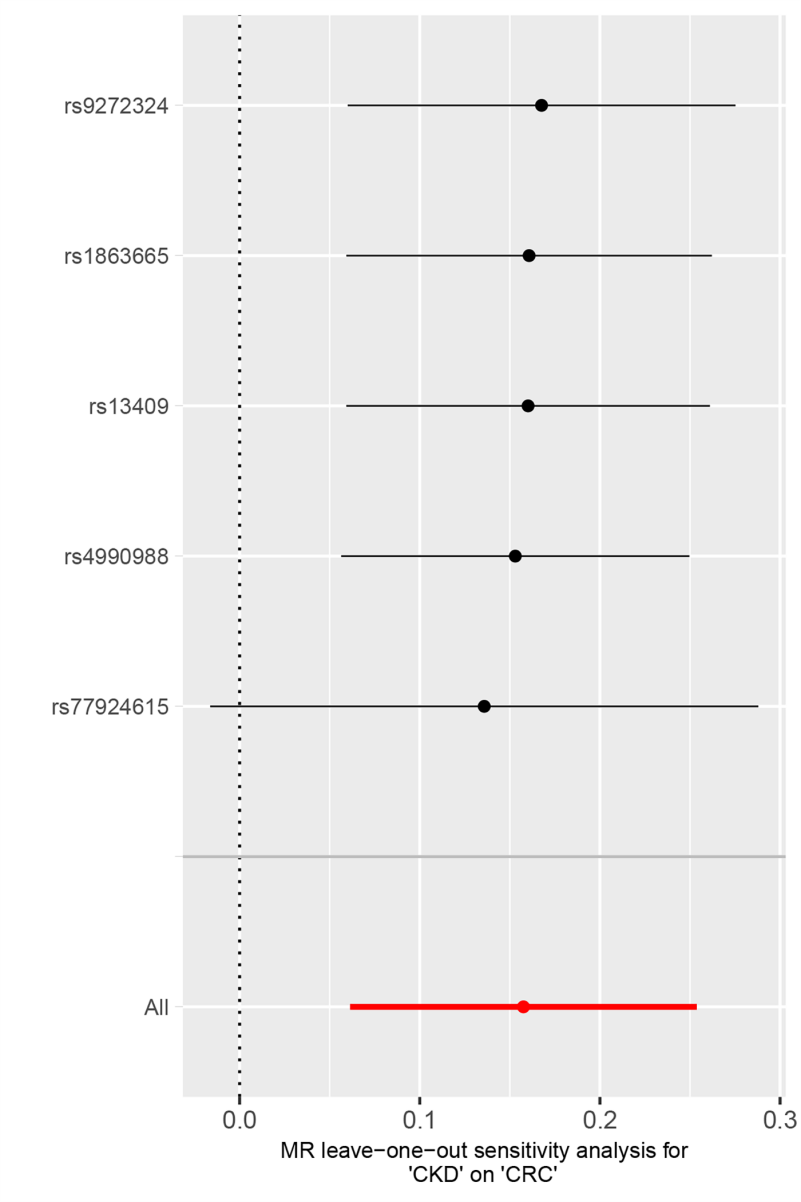


**Fig. S10** Funnel plots of the results from MR analysis of chronic kidney disease on colorectal cancer.


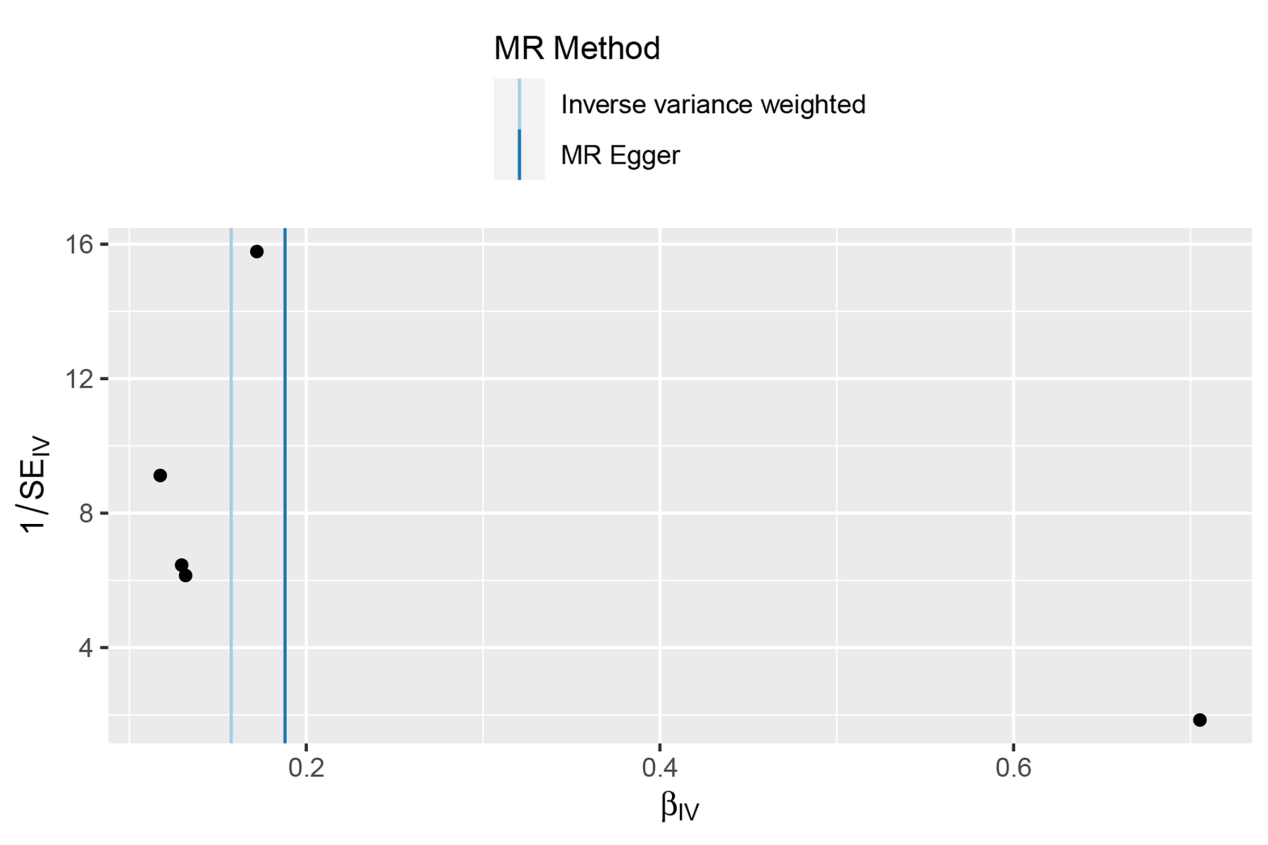

Supplement: Supplementary file 1 — Supplementary material 1: Fig. S1 Study design diagram and three assumptions of Mendelian randomization. SNPs, single nucleotide polymorphisms; LD, linkage disequilibrium. Fig. S2 Subgroup analysis of the association between chronic kidney disease and colorectal cancer based the race of participants (Subgroup = East Asian). Fig. S3 Subgroup analysis of the association between chronic kidney disease and colorectal cancer based on the age of participants. A < 50 years. B ≥ 50 years. Fig. S4 Subgroup analysis of the association between chronic kidney disease and colorectal cancer based on the gender of participants. A Male. B Female. Fig. S5 Subgroup analysis of the association between chronic kidney disease and colorectal cancer based on the dialysis type. A Hemodialysis. B Peritoneal dialysis. Fig. S6 Sensitivity analysis of the association between chronic kidney disease and colorectal cancer. Fig. S7 Funnel plot of the results from meta-analysis of the association between chronic kidney disease and colorectal cancer. Fig. S8 Forest plots of the results from MR analysis of chronic kidney disease on colorectal cancer. Fig. S9 Leave-one-out analysis of the results from MR analysis of chronic kidney disease on colorectal cancer. Fig. S10 Funnel plots of the results from MR analysis of chronic kidney disease on colorectal cancer. [file 12672_2025_2785_MOESM1_ESM.docx]
