# Supplementary material for: Association between chronic kidney disease and colorectal cancer: evidence from meta-analysis and Mendelian randomization
Source: Discov Oncol. 2025 Jun 1;16:974. doi: 10.1007/s12672-025-02785-9 (PMC12127261; doi:10.1007/s12672-025-02785-9)
Supplement: Supplementary file 2 — Supplementary material 2. [file 12672_2025_2785_MOESM2_ESM.docx]

| **PubMed 755** |
| --- |
| #1 (chronic kidney disease) OR (CKD) OR (chronic renal disease) OR (chronic kidney insufficiency) OR (chronic renal insufficiency) OR (chronic kidney failure) |
| #2 (colorectal cancer) OR (colon cancer) OR (rectal cancer) OR (colorectal tumor) OR (colorectal neoplasm) OR (colorectal carcinoma) |
| #3 #1 AND #2 |
| **Web of Science 716** |
| #1 TS=((chronic kidney disease) OR (CKD) OR (chronic renal disease) OR (chronic kidney insufficiency) OR (chronic renal insufficiency) OR (chronic kidney failure)) |
| #2 TS=((colorectal cancer) OR (colon cancer) OR (rectal cancer) OR (colorectal tumor) OR (colorectal neoplasm) OR (colorectal carcinoma)) |
| #3 #1 AND #2 |
| **The Cochrane Library 272** |
| #1 All Text=((chronic kidney disease) OR (CKD) OR (chronic renal disease) OR (chronic kidney insufficiency) OR (chronic renal insufficiency) OR (chronic kidney failure)) |
| #2 All Text=((colorectal cancer) OR (colon cancer) OR (rectal cancer) OR (colorectal tumor) OR (colorectal neoplasm) OR (colorectal carcinoma)) |
| #3 #1 AND #2 |
| **Embase 360** |
| #1 'chronic kidney disease':ti,ab,kw OR ckd:ti,ab,kw OR 'chronic renal disease':ti,ab,kw OR 'chronic kidney insufficiency':ti,ab,kw OR 'chronic renal insufficiency':ti,ab,kw OR 'chronic kidney failure':ti,ab,kw |
| #2 'colorectal cancer':ti,ab,kw OR 'colon cancer':ti,ab,kw OR 'rectal cancer':ti,ab,kw OR 'colorectal tumor':ti,ab,kw OR 'colorectal neoplasm':ti,ab,kw OR 'colorectal carcinoma':ti,ab,kw |
| #3 #1 AND #2 |
