# Supplementary material for: Association between chronic kidney disease and colorectal cancer: evidence from meta-analysis and Mendelian randomization
Source: Discov Oncol. 2025 Jun 1;16:974. doi: 10.1007/s12672-025-02785-9 (PMC12127261; doi:10.1007/s12672-025-02785-9)
Supplement: Supplementary file 3 — Supplementary material 3. [file 12672_2025_2785_MOESM3_ESM.docx]

| **Table S1** The GWAS data source details in our Mendelian randomization study. | | | | | | | |
| --- | --- | --- | --- | --- | --- | --- | --- |
| Phenotype | Cases | Controls | Sample size | nSNP | Ethnicity | PMID | Data source |
| **Exposure** | | | | | | | |
| Chronic kidney disease | 11,265 | 436,208 | 447,473 | 21,306,707 | European | NA | FinnGen |
| **Outcome** | | | | | | | |
| Colorectal cancer | 6,581 | 463,421 | 470,002 | 24,182,361 | European | 34594039 | IEU Open GWAS |
| NA, not available. | | | | | | | |

| Table S2 The Newcastle-Ottawa quality assessment scale of the included cohort studies. | | | | | | | | | | | | |
| --- | --- | --- | --- | --- | --- | --- | --- | --- | --- | --- | --- | --- |
| Study | Selection | | | |  | Comparability | |  | Assessment of outcome | | | Total score |
|  | Representativeness of exposure arm(s) | Selection of the comparative arm(s) | Origin of exposure source | Demonstration that outcome of interest was not present at start of study |  | Studies controlling the most important factors | Studies controlling the other main factors |  | Assessment of outcome with independency | Adequacy of follow-up length | Lost to follow-up acceptable |  |
| Kwon (2019) | 1 | 1 | 1 | 1 |  | 1 | 0 |  | 1 | 1 | 1 | 8 |
| Lee (2018) | 1 | 1 | 1 | 1 |  | 1 | 0 |  | 1 | 0 | 1 | 7 |
| Lin (2015) | 1 | 1 | 1 | 1 |  | 1 | 0 |  | 1 | 1 | 1 | 8 |
| Wu (2013) | 1 | 1 | 1 | 1 |  | 1 | 0 |  | 1 | 1 | 1 | 8 |
| Park (2019) | 1 | 1 | 1 | 1 |  | 1 | 0 |  | 1 | 1 | 1 | 8 |
| Jung (2022) | 1 | 1 | 1 | 1 |  | 1 | 0 |  | 1 | 1 | 1 | 8 |
| Chung (2012) | 1 | 1 | 1 | 1 |  | 1 | 0 |  | 1 | 1 | 1 | 8 |
| Wang (2019) | 1 | 1 | 1 | 1 |  | 1 | 0 |  | 1 | 0 | 1 | 7 |
